# Factor Structures of Patient Health Questionnaire-9 Instruments in Exploring Depressive Symptoms of Suburban Population

**DOI:** 10.3389/fpsyt.2022.838747

**Published:** 2022-08-03

**Authors:** Linh Gia Vu, Linh Khanh Le, Anh Vu Trong Dam, Son Hoang Nguyen, Thuc Thi Minh Vu, Trang Thu Hong Trinh, Anh Linh Do, Ngoc Minh Do, Trang Huyen Le, Carl Latkin, Roger C. M. Ho, Cyrus S. H. Ho

**Affiliations:** ^1^Institute for Global Health Innovations, Duy Tan University, Da Nang, Vietnam; ^2^Faculty of Medicine, Duy Tan University, Da Nang, Vietnam; ^3^Troy University, Troy, AL, United States; ^4^Center of Excellence in Evidence-Based Medicine, Nguyen Tat Thanh University, Ho Chi Minh City, Vietnam; ^5^Institute of Health Economics and Technology, Hanoi, Vietnam; ^6^Harvard Extension School, Harvard University, Cambridge, MA, United States; ^7^Vinschool, Hanoi, Vietnam; ^8^Sub-Department of Food Hygiene and Safety, Hanoi, Vietnam; ^9^Institute for Preventive Medicine and Public Health, Hanoi Medical University, Hanoi, Vietnam; ^10^Bloomberg School of Public Health, Johns Hopkins University, Baltimore, MD, United States; ^11^Department of Psychological Medicine, Yong Loo Lin School of Medicine, National University of Singapore, Singapore, Singapore; ^12^Institute for Health Innovation and Technology (iHealthtech), National University of Singapore, Singapore, Singapore

**Keywords:** validation, reliability, depression, PHQ9, suburban area

## Abstract

**Background:**

This study aims to examine the psychometric properties of the nine-item Patient Health Questionnaire (PHQ-9) and assess the relationship between the PHQ-9 domain and demographics and health behaviors in Vietnamese people.

**Materials and Methods:**

The PHQ9 was administered to 899 participants. Exploratory factor and reliability analyses were performed. Tobit regression and Ordered logistic regression were further performed to determine factors associated with the PHQ-9 score and characteristics of depression.

**Results:**

The 2-factor model of PHQ-9, including factor 1 “Somatic” and factor 2 “Cognitive/Affective,” showed good psychometric properties. The Cronbach’s alpha value showed high internal consistency in two factors (0.84 and 0.80, respectively). Gender, health behavior exercising, drinking, and health status had associations with both factors of the PHQ-9 model.

**Conclusion:**

The PHQ-9 scale is a valid and reliable instrument to assess depression in the Vietnam population. This scale can be a useful screening tool for depression; however, further validation studies in other populations are required.

## Introduction

One of the most common mental illnesses in the world is depression (1). Depression is a mental condition characterized by chronic sorrow and a loss of interest. It affects how you feel, think, and behave and can lead to several mental and physical difficulties. It’s also known as major depressive disorder or clinical depression (2, 3). In 2017, 4.4% of the global population suffered from depression, according to the World Health Organization (1). Furthermore, depression is the leading cause of disability, is frequently associated with an increased mortality rate since depressed people are much more likely to commit suicide (4, 5). The urbanization process is among the influencing environmental factors that can cause mental disorders (6–8). Several studies have also shown that mental health issues are likely to unfold in countries with higher rates of urbanization due to their associated socio-demographic transitions (6, 9–11). Individuals living in these areas must adjust to new fast-paced lifestyles, and face intense competition for jobs, commodities, goods, and services, and thus potentially experience increased stress levels (6, 12). Furthermore, urbanization comes with the creation of communities comprising of people who may not know each other well, such communities can undermine friendship, familial connections, and diminish traditional social solidarity, and as a consequence, the local social capital is weakened in the metropolitan setting (9, 13). Understanding mental disorder patterns and identifying appropriate measuring tools thus are necessary for the process of designing contextualized interventions that help improve the well-being state of people living in areas with accelerated urbanization.

Although there is a variety of available instruments to assess depressive symptoms, the nine-item Patient Health Questionnaire (PHQ-9), extensively utilized assessments in multiple research and countries, is one of the most commonly used tools for diagnosis and severity assessment of depression (14–17). It can be used either as a diagnostic algorithm to make a probable diagnosis of major depressive disorder (MDD) or as a continuous measure of the level of depressive symptoms. The PHQ-9 measures the severity of depression by assessing symptoms such as depressed mood, sleep disturbance, fatigue, concentration problems, psychomotor disturbances, and suicidal ideation following the Diagnostic and Statistical Manual for Mental Disorders, Fourth Edition (DSM-IV-TR) (18, 19). Furthermore, the PHQ-9’s psychometric qualities have been tested on various populations, namely mental patients (20, 21), groups of medical patients (22, 23), the elderly (24), general adult population (25, 26). Although the PHQ-9’s internal consistency was demonstrated to be adequate (α = 0.70–0.93) (20–26) and the results of this scale showed moderate-to-strong relationships with the associated measures of depression, investigations of PHQ-9’s underlying component structure were often inconsistent. Several studies revealed that a one-factor model representing the one-dimensionality of the depression construct provided the best fit to the data among different demographic groups (1, 18, 26–28). Nonetheless, other research discovered that the PHQ-9 factor structure was best represented by two-factor models that recognize both somatic and cognitive/affective (20–23, 29). Because of this difference in previous research findings, it is critical to determine whether the PHQ-9 model is the most helpful in explaining depression using different local populations.

In Vietnam, there have been numerous studies on the factors associated with depressive disorder in various groups, such as the elderly, pregnant women, workers, etc. (30–32); however, none of them determined the reliability and tested the validity of PHQ-9 in the community context (30–32). As a result, the goal of this research is to provide data on the psychometric aspects of PHQ9, such as reliability and validity and to determine influencing factors that may affect depression assessment in the community context.

## Materials and Methods

### Study Design and Participants

A cross-sectional study was conducted in three communes of Thanh Oai District, Hanoi, Vietnam: Lien Chau, Thanh Van, and Tam Hung in July 2019. The eligibility criteria for participating in this study include (1) being 18 years old or above; (2) living in one of the above-mentioned communes, and (3) agreeing to participate in the study. Participants who were unable to answer or complete the questionnaire were excluded from the study. A convenience sampling approach was performed to recruit participants. A total of 889 persons consented to participate in the study from a list of residents in three communes with the help of local health professionals.

### Measure and Instruments

Participants were asked to go to nearby community health centers for a 30-min face-to-face interview with well-trained local health professionals and medical staff. The objectives of the study, as well as the benefits and rights of participants, were briefly explained to them. Once agreed to participate in the study, participants signed a written informed consent in which their confidentiality was guaranteed. The collected information from the structured questionnaire provided during the interviews with participants is described below.

#### Primary Outcome

*Patient Health Questionnaire-9 (PHQ-9)* was used to measure the levels of depression among participants, based on the Diagnostic and Statistical Manual of Mental Disorders (DSM-IV) criteria. There are nine questions, answers to which were graded on a scale from 0 to 3. The total score ranged from 0 to 27. The intensity of depression was classified as none (0–4 points), mild (5–9 points), moderate (10–14 points), fairly severe (15–19 points), and severe (20 points). For severe depression, based on previous study, a PHQ-9 score of 5 was considered as the cut-off point for our study (14, 16, 33). The Cronbach’s alpha of PHQ-9 was 0.85.

#### Predictor Variables

##### Socio-Economic Characteristics

Participants were asked to self-report their living location, gender, educational level, marital status, occupation, health insurance, number of family members, age, and total household monthly income. The household monthly income was given in US dollars, with 22,955 VND equaling 1 US dollar at the 2019 conversion cost. The income was also divided into three quintiles from low to high.

##### Health Status

Data related to health status were collected from the self-reports of participants. Collected data included their number of health problems and their first-choice institution for treatment when experiencing health issues.

The participants’ quality of life was assessed using *the EuroQOL-5 Dimensions 5 level (EQ-5D-5L)*. The EQ-5D-5L was a weighted average of five dimensions: (1) Mobility, (2) Self-care, (3) Usual activities, (4) Pain/Discomfort, and (5) Anxiety/Depression. For each dimension, the score ranged from 1 (no problems) to 5 (severe problem) (34). Participants were assigned to the category “Having problems” if they chose any answers from slight problems to severe problems. The Vietnamese version of the EQ-5D-5L was applied in this study (35).

##### Health Behavior

Participants were asked to report their current smoking behavior (yes/no) and current level of alcohol consumption (yes/no).

Participants’ leisure-time physical activities were assessed by using the Godin Leisure-time exercise questionnaire, which was chosen due to its quick and practical nature. Individual physical activity/exercise score in this questionnaire was calculated based on the frequency and type of activities/exercises they did weekly. Godin Leisure-time exercise = strenuous (number of strenuous activities per week × 3) + moderate (number of moderate activities per week × 6) + light (number of light activities per week × 14). The Godin Leisure-time exercise score was later categorized into three levels: Insufficiently Active/Sedentary (less than 14 points), Moderately Active (14–23 points), and Active (24 points or more) (36). The Cronbach’s alpha of the Godin Leisure-time exercise questionnaire was 0.79.

##### Health Information Source

Participants reported their health information sources, such as friends/relatives, mass media, radio/television, or health care workers.

### Statistical Analysis

The data were analyzed using STATA 15.0 (StataCorp LP, College Station, TX, United States). The qualitative variables were evaluated by assessing the frequency and percentage, whereas the quantitative variables were studied by analyzing the mean and standard deviation (SD). The Chi-square and Kruskal Wallis tests were used to compare the differences in socioeconomic position, health status, habits, and health care usage between those who exhibited depressive symptoms and those who did not.

We used Ordered logistic regression to determine factors associated with the PHQ-9 score and characteristics of depression. Besides, the Tobit regression, also called a censored regression model, was also used to estimate linear relationships between the variables when there is either left- or right-censoring in the outcome variables. In addition, multivariate logistic regression was employed to identify the potential factors associated with the presence of depressive symptoms among participants. Moreover, we utilized a forward stepwise selection strategy, which included variables having *p*-values of <0.2 in the log-likelihood ratio tests, together with regression models to construct a reduced model. A *p*-value of less than 0.05 was considered statistically significant.

#### Reliability

Internal consistency dependability was measured using Cronbach’s alpha. An alpha value of 0.7 or above was considered acceptable (37). In addition, we would calculate the domain-domain correlation, item-item correlation, item-total correlation, Coefficient omega, and Cronbach’s alpha of the domain.

#### Factorial Structure

**FIGURE 1:**
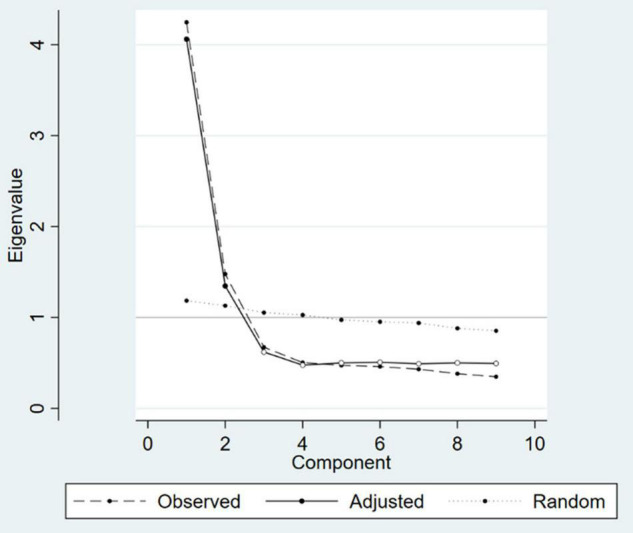
Scree plot and parallel analysis.

Based on the observed data, exploratory factor analysis (EFA) with principal component analysis (PCA) was used to establish the optimum structural model for the chosen study instrument. The scree plot and parallel analysis (Figure 1) were used to identify the number of components, as well as eigenvalues and the amount of variance explained (38). Items with a loading value ≥0.35 were considered to be included in the relevant component (38).

Then, we performed Confirmatory Factor Analysis (CFA) to see if the previous model (1 factor) of PHQ-9 and the revised model (2 factors) could explain depression better. In order to test the model fit of the observed data, many model fit indicators with respective cut-offs were explored (using Satorra-Bentler correction for non-normality data), including (39):

• Relative Chi-square (χ^2^/df): a value ≤3.0 for good fit.
• Root Mean Square Error of Approximation (RMSEA): a value of ≤0.08 for good fit.
• Comparative Fit Index (CFI): a value of ≥0.9 for acceptable fit.
• Standardized Root Mean Square Residual (SRMR): a value of ≤0.08 for good fit.
• Akaike’s Information Criteria (AIC) and Bayesian Information Criteria (BIC): the model with lower scores of AIC and BIC would be chosen.

### Ethical Consideration

All participants provided written informed consent. The data, which was kept in safe places, was only accessible to the principal investigators. This research project was reviewed and managed by the Vietnam Ministry of Health under Decision 850/QD-BYT. Ethical approval was granted by the IRB committee at Hanoi Medical University and the Ministry of Health.

## Results

**FIGURE A1:**
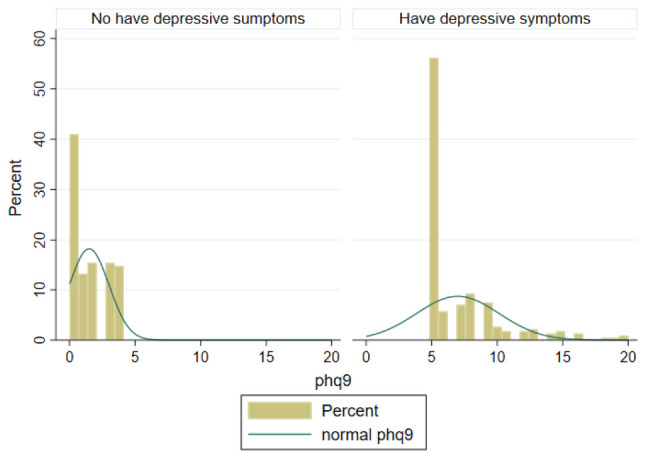
The distribution of Patient Health Questionnaire (PHQ-9) scores by depressive symptoms.

Table 1 summarizes the sociodemographic characteristics of participants. 25.7% of 889 respondents to the survey had at least one of the depressive symptoms. The majority of participants were women (58.9%), lived in rural areas (86.8%), and had primary/secondary school education levels (33.5%). 26.8% of respondents were farmers and 25.2% were freelancers. The mean age of respondents was 42.12 (SD = 13.38), and the average household monthly income was US$ 433.12 (SD = US$ 241.54). The distribution of PHQ-9 scores among depressed and non-depressed patients was described in Appendix Figure A1.

**TABLE 1:** Sociodemographic characteristics of respondents.

Characteristics	Depressive symptoms						*P*-value^1^
			
	No		Yes		Total		
				
	*n*	%	*n*	%	*n*	%	
Total	661	74.4	228	25.7	889	100.0	
**Living area**							
Urban	74	11.3	43	18.9	117	13.2	0.004
Rural	582	88.7	185	81.1	767	86.8	
**Gender**							
Male	276	42.0	88	38.6	364	41.1	0.376
Female	382	58.1	140	61.4	522	58.9	
**Education**							
Not go to school	10	1.5	3	1.3	13	1.5	0.031
Primary/Secondary school	216	32.7	81	35.7	297	33.5	
High school	201	30.5	48	21.2	249	28.1	
College	155	23.5	54	23.8	209	23.6	
University/Post-graduate	78	11.8	41	18.1	119	13.4	
**Marital status**							
Single/Divorce/Widow	86	13.0	26	11.4	112	12.6	0.528
Have spouse/partner	575	87.0	202	88.6	777	87.4	
**Occupation**							
Job without income	31	4.7	9	4.0	40	4.5	0.005
Freelancer	171	25.9	53	23.3	224	25.2	
White-collar	124	18.8	55	24.1	179	20.1	
Worker	85	12.9	32	14.0	117	13.2	
Farmer	168	25.4	70	30.7	238	26.8	
Other	82	12.4	9	4.0	91	10.2	
**Monthly income quintile**							
Low	267	41.0	94	41.4	361	41.1	0.376
Medium	174	26.7	51	22.5	225	25.6	
High	210	32.3	82	36.1	292	33.3	
**Health insurance**							
No	190	29.1	37	16.3	227	25.8	<0.001
Yes	464	71.0	190	83.7	654	74.2	
**Leisure-time exercise**							
Sedentary	234	35.9	71	31.6	305	34.8	0.009
Moderately	98	15.0	54	24.0	152	17.3	
Active	320	49.1	100	44.4	420	47.9	
	**Mean**	**SD**	**Mean**	**SD**	**Mean**	**SD**	***p*-value**
Age (unit: year)	41.18	12.74	44.86	14.80	42.12	13.38	0.003
Number of family member	4.10	1.24	3.73	1.47	4.00	1.31	<0.001
Household monthly income (unit: dollar)	428.60	212.31	446.08	310.69	433.12	241.54	0.754

^1^The p-value refers to test the statistical differences between people with depressive symptoms and those without.

Table 2 shows the health status and risk behaviors of the respondents. More than half of the participants reported at least one symptom indicating health problems (56.8%). The proportion of participants who consumed alcohol and those who smoked were 34.0 and 19.3%, respectively. Healthcare workers (72.5%), friends/relatives (56.4%), and radio/television (43.3%) were the most preferred sources of health information for participants. The mean EQ5D score was 0.92 (SD = 0.12), while the average PHQ-9 score was 2.91 (SD = 3.16). There was a statistical significance of the EQ5D and PHQ-9 scores that differed between depressed and non-depressed patients (*p*-value < 0.001). The effect size (Cohen’s *d*) from the EQ5D and PHQ-9 was 0.85 (95% CI = 0.70–1.01) and 2.67 (95% CI = 2.47–2.86), which indicates a large effect size.

**TABLE 2:** Health status and risk behaviors of respondents.

Characteristics	Depression symptoms						*P*-value^1^
			
	No		Yes		Total		
				
	*n*	%	*n*	%	*n*	%	
**Drinking alcohol**							
No	442	67.1	143	63.0	585	66.0	0.263
Yes	217	32.9	84	37.0	301	34.0	
**Smoking**							
No	496	81.9	154	77.4	650	80.8	0.166
Yes	110	18.2	45	22.6	155	19.3	
**Number of health problem**							
No health problem	331	50.1	53	23.3	384	43.2	<0.001
1 health problem	293	44.3	137	60.1	430	48.4	
2 health problem and more	37	5.6	38	16.7	75	8.4	
**Institution of first choice**							
Central/Province hospital	95	14.4	26	11.5	121	13.7	<0.001
District hospital	66	10.0	53	23.5	119	13.5	
Community health center	394	59.8	82	36.3	476	53.8	
Private Clinic	51	7.7	45	19.9	96	10.9	
Self-treatment	53	8.0	20	8.9	73	8.3	
**Health information**							
Friends/relatives	389	58.9	111	49.3	500	56.4	0.013
Poster/banner	39	5.9	18	8.0	57	6.4	0.270
Internet	266	40.2	88	39.1	354	40.0	0.765
Messages	34	5.2	11	4.9	45	5.1	0.877
Radio/television	321	48.7	62	27.4	383	43.3	<0.001
Speaker	125	18.9	93	41.3	218	24.6	<0.001
Newspaper	92	13.9	17	7.6	109	12.3	0.012
Health care worker	476	72.2	165	73.3	641	72.5	0.749
Social network	264	40.0	63	28.0	327	37.0	0.001
	**Mean**	**SD**	**Mean**	**SD**	**Mean**	**SD**	***p*-value**
PHQ9 Score (unit: score)	1.50	1.51	7.00	3.16	2.91	3.16	<0.001
EQ-index (unit: score)	0.94	0.10	0.85	0.16	0.92	0.12	<0.001

^1^The p-value refers to test the statistical differences between people with depressive symptoms and those without.

**FIGURE 2:**
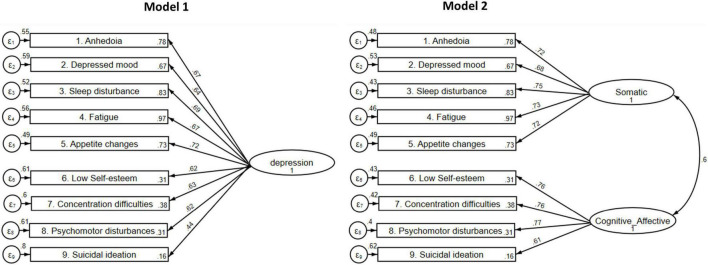
Evaluated Patient Health Questionnaire (PHQ-9) factor structures. *Model 1:* One-factor PHQ9 model (Chi-Square = 749.019; df = 27; TLI = 0.697; CFI = 0.773; RMSEA (90%CI) = 0.174 (0.163–0.184); SRMR = 0.102; *p*-value ≤ 0.001; AIC = 8371.595; BIC = 8500.928); *Model 2:* two-correlated factor PHQ9 model (Chi-Square = 152.183; df = 26; TLI = 0.945; CFI = 0.960; RMSEA (90%CI) = 0.074 (0.063–0.086); SRMR = 0.043; *p*-value ≤ 0.001; AIC = 7776.759; BIC = 7910.882). df, degrees of freedom; TLI, Tucker-Lewis Index; CFI, comparative fit index; RMSEA, root-mean-square error of approximation; RMSEA 90% CI: 90% confidence interval for RMSEA; SRMR: standardized root-mean-square residual.

Two factors, namely Somatic (five items) and Cognitive or Affective (four items), were identified using the scree plot and parallel analysis (Figure 1) and exploratory factor analysis, with Cronbach’s alphas of 0.84 and 0.80, respectively (Table 3). Furthermore, our CFA results revealed that the model 2 with two factors had better fit indices [TLI = 0.945; CFI = 0.960; RMSEA (90%CI) = 0.074 (0.063–0.086); SRMR = 0.043; *p*-value = < 0.001] compared to the original instrument (Model 1) [TLI = 0.697; CFI = 0.773; RMSEA (90%CI) = 0.174 (0.163–0.184); SRMR = 0.102; *p*-value = < 0.001; Figure 2].

**TABLE 3:** Exploratory factor analysis for Patient Health Questionnaire (PHQ-9) instrument.

Variable	Factor 1: Somatic	Factor 2: Cognitive/Affective
Anhedonia	**0.682**	0.201
Depressed mood	**0.645**	0.210
Sleep disturbance	**0.715**	0.199
Fatigue	**0.708**	0.168
Appetite changes	**0.622**	0.341
Low Self-esteem	0.270	**0.703**
Concertation difficulties	0.299	**0.666**
Psychomotor disturbances	0.263	**0.701**
Suicidal ideation	0.088	**0.637**
Cronbach’s alpha	0.843	0.802
Coefficient omega	0.844	0.820

This highlight the higher value of each variable.

Table 4 shows the results of the descriptive analysis for each PHQ-9 item. All of the nine items had a range of scores from 0 to 3. The Skewness and Kurtosis coefficients ranged from 0.97–6.71 to 3.84–51.76, respectively. Nine out of nine items had Kurtosis coefficients greater than 3.0, indicating that these data distribution had long and fat tails as well as high and shape peaks. The mean and standard deviation of each item in PHQ-9 scale were also reported in this table. Moreover, most of the nine items showed high correlation coefficients with other items in the respective factor (*r* > 0.6).

**TABLE 4:** Basic descriptions and reliability of Patient Health Questionnaire (PHQ-9) instrument.

Items	Respondence (%)				Mean (SD)	Skewness	Kurtosis	Item-total correlation	Cronbach’s alpha if item deleted
						
	Not at all	Some	Often	Nearly all of the time					
Factor 1: Somatic					0.49 (0.48)	1.02	4.17		
Anhedonia	515 (57.93)	334 (37.57)	35 (3.94)	5 (0.56)	0.47 (0.60)	1.04	3.84	0.65	0.81
Depressed mood	588 (66.14)	275 (30.93)	22 (2.47)	4 (0.45)	0.37 (0.56)	1.34	4.67	0.62	0.82
Sleep disturbance	476 (53.54)	362 (40.72)	41 (4.61)	10 (1.12)	0.53 (0.64)	1.05	4.15	0.68	0.80
Fatigue	390 (43.87)	438 (49.27)	42 (4.72)	19 (2.14)	0.65 (0.67)	0.97	4.41	0.67	0.81
Appetite changes	546 (61.42)	303 (34.08)	35 (3.94)	5 (0.56)	0.44 (0.60)	1.19	4.16	0.63	0.82
Factor 2: Cognitive/Affective					0.11 (0.29)	3.93	22.47		
Low Self-esteem	796 (89.54)	80 (9.00)	7 (0.79)	6 (0.67)	0.13 (0.41)	3.98	22.05	0.66	0.73
Concertation difficulties	761 (85.60)	114 (12.82)	9 (1.01)	5 (0.56)	0.17 (0.44)	3.11	14.67	0.66	0.74
Psychomotor disturbances	799 (89.88)	79 (8.89)	8 (0.90)	3 (0.34)	0.12 (0.38)	3.77	19.99	0.68	0.72
Suicidal ideation	863 (97.08)	21 (2.36)	5 (0.56)	0 (0.00)	0.03 (0.21)	6.71	51.76	0.57	0.80
PHQ-9 score (range: 0–27)					2.91 (3.16)	1.78	7.73		

Table 5 summarizes the factor of the PHQ-9 instrument score based on various socio-economic characteristics. The mean Somatic factor and Cognitive/Affective factor scores were 0.49 (SD = 0.48) and 0.11 (SD = 0.29), with the statistically higher score for people living in urban areas. Notable differences in these factors in terms of living location, level of education, and leisure-time exercise were observed (*p* < 0.01).

**TABLE 5:** Patient Health Questionnaire (PHQ-9) - factors scores by different characteristics.

Characteristics	Factor 1: Somatic			Factor 2: Cognitive/Affective		
		
	Mean	SD	*P*-value	Mean	SD	*P*-value
Total	0.49	0.48		0.11	0.29	
**Living area**						
Urban	0.72	0.60	<0.001	0.19	0.34	<0.001
Rural	0.46	0.45		0.10	0.28	
**Gender**						
Male	0.45	0.46	0.043	0.11	0.29	0.536
Female	0.52	0.50		0.11	0.29	
**Education**						
Not go to school	0.68	0.57	0.027	0.13	0.24	0.002
Primary/Secondary school	0.50	0.46		0.09	0.26	
High school	0.42	0.45		0.09	0.26	
College	0.49	0.46		0.11	0.30	
University/Post-graduate	0.61	0.58		0.20	0.39	
**Marital status**						
Single/Divorce/Widow	0.45	0.51	0.144	0.16	0.38	0.052
Have spouse/partner	0.50	0.48		0.10	0.28	
**Occupation**						
Job without income	0.50	0.54	0.213	0.09	0.20	0.022
Freelancer	0.43	0.46		0.13	0.32	
White-collar	0.54	0.56		0.15	0.36	
Worker	0.50	0.46		0.08	0.21	
Farmer	0.52	0.47		0.10	0.28	
Other	0.49	0.39		0.06	0.22	
**Monthly income quintile**						
Low	0.51	0.48	0.200	0.11	0.31	0.600
Medium	0.44	0.45		0.11	0.31	
High	0.51	0.51		0.11	0.26	
**Health insurance**						
No	0.46	0.43	0.508	0.07	0.18	0.052
Yes	0.50	0.50		0.13	0.32	
**Leisure-time exercise**						
Sedentary	0.47	0.51	<0.001	0.11	0.29	0.001
Moderately	0.63	0.42		0.04	0.16	
Active	0.46	0.47		0.14	0.33	

Table 6 indicates the factors associated with depression of participants. The PHQ-9 score of older participants was statistically higher, as was the somatic factor. While female participants had a statistically higher PHQ-9 score and were more prone to experience depressive symptoms and somatic characteristics, those residing in rural regions had a statistically lower score. In terms of health behavior and health status, people with health issues had statistically higher PHQ-9 scores and were more likely to experience depressive symptoms; somatic factors; or cognitive/affective factors. Those who engaged in moderate leisure-time exercise had a statistically significant association with lower cognitive/affective variables, whereas those who engaged in heavy drinking were more inclined to have depressive symptoms, somatic factors, and a higher PHQ-9 score. Individuals who obtained health information via radio/television, newspaper, health care workers, or social networks were less likely to suffer from depression in comparison to those who listened through the loudspeaker.

**TABLE 6:** Regression results of factors associated with depression among participants.

	PHQ-9 Score		Depressive symptoms		PHQ-9_Somatic		PHQ-9_Cognitive/Affective	
				
	Coef.	95% CI	OR	95% CI	Coef.	95% CI	Coef.	95% CI
**Social-Economic**								
**Living area (vs Urban)**								
Rural	−1.06**	−1.98; −0.13	0.57**	0.33; 0.99	−0.20***	−0.33; −0.06	–0.17	−0.45; 0.10
**Gender (vs Male)**								
Female	1.74***	0.77; 2.72	3.78***	1.90; 7.52	0.33***	0.18; 0.48		
Age (unit: year)	0.04***	0.01; 0.06			0.01***	0.00; 0.01		
**Education (vs Not going to school)**								
Primary/Secondary school	−1.37	−4.12; 1.38			–0.17	−0.58; 0.24	–0.57	−1.33; 0.20
High school	−0.81	−3.57; 1.94			–0.11	−0.52; 0.30	–0.42	−1.19; 0.35
College	−0.30	−3.08; 2.48			0.01	−0.41; 0.43	–0.16	−0.94; 0.63
University/Post-graduate	0.27	−2.57; 3.10			0.10	−0.33; 0.54	–0.01	−0.81; 0.80
**Occupation (vs Job without income)**								
Freelancer			2.31	0.78; 6.83	–0.03	−0.27; 0.21	0.16	−0.33; 0.64
White-collar			1.61	0.55; 4.71	–0.13	−0.38; 0.12	–0.14	−0.64; 0.36
Worker			1.45	0.47; 4.48	–0.01	−0.26; 0.25	–0.19	−0.71; 0.33
Farmer			1.43	0.48; 4.25	–0.08	−0.34; 0.18	–0.08	−0.59; 0.43
Other			0.74	0.20; 2.78	0.10	−0.17; 0.38	−0.57*	−1.16; 0.02
**Household monthly income quintile (vs Low)**								
Medium					−0.09*	−0.20; 0.02		
High					–0.08	−0.18; 0.03		
**Health insurance (vs No)**								
Yes			1.67**	1.01; 2.75				
**Health behavior and health status**								
**Drinking (vs No)**								
Yes	1.13**	0.12; 2.13	3.49***	1.75; 7.00	0.16*	−0.00; 0.31		
**Smoking (vs No)**								
Yes					0.09	−0.04; 0.21		
**Leisure-time exercise (vs Sedentary)**								
Moderately							−0.56***	−0.90; −0.23
Active							0.02	−0.20; 0.24
**Number of health problems (vs No health problem)**								
1 health problem	1.90***	1.24; 2.55	2.49***	1.59; 3.89	0.32***	0.22; 0.42	0.24**	0.02; 0.47
2 health problem and more	3.16***	2.07; 4.26	6.33***	3.29; 12.20	0.51***	0.35; 0.67	0.50***	0.16; 0.84
**Institution of first choice (vs Central/Province hospital)**								
District hospital	1.09*	−0.02; 2.20	1.65	0.80; 3.41	0.20**	0.03; 0.36	0.08	−0.28; 0.43
Community health center	−0.87*	−1.80; 0.06	0.62	0.33; 1.18	–0.10	−0.24; 0.03	−0.25*	−0.54; 0.05
Private Clinic	0.70	−0.51; 1.92	2.45**	1.13; 5.29	0.22**	0.03; 0.40	−0.57**	−1.02; −0.12
Self-treatment	−0.24	−1.51; 1.02	0.78	0.34; 1.78	0.00	−0.18; 0.19	–0.00	−0.38; 0.38
**Source of health information (Yes vs No)**								
Friends/relatives	−0.46	−1.06; 0.13			–0.07	−0.16; 0.02	–0.16	−0.36; 0.04
Radio/television	−1.39***	−2.04; −0.75	0.52***	0.34; 0.81	−0.25***	−0.35; −0.15		
Speaker	1.02***	0.31; 1.74	2.69***	1.73; 4.19	0.20***	0.09; 0.30		
Newspaper	−0.68	−1.61; 0.26	0.37***	0.18; 0.76	−0.14*	−0.28; 0.00		
Health care worker	−0.52	−1.20; 0.15	0.73	0.47; 1.15			−0.25**	−0.46; −0.04
Social network	−1.24***	−1.88; −0.60	0.62**	0.40; 0.96	−0.15***	−0.25; −0.06	−0.40***	−0.62; −0.18
*n*	769		769		769		769	
Pseudo R^2^	0.072***		0.182***		0.224***		0.201***	

***p < 0.01; **p < 0.05; *p < 0.1.

## Discussion

The results of the study reveal that those individuals who were females and residents of urban areas had a higher prevalence of depressive symptoms. Associations between level of depressive symptoms and individual characteristics, such as age, gender, health status, drinking behavior, level of physical activity, and access to information sources were also found. Furthermore, the study provided insights into the measuring features of the PHQ-9 instrument in assessing depression for the general population in Vietnam. The two-factor model, which includes factor 1 “Somatic” and factor 2 “Cognitive/Affective,” was shown to have better fit indices than the theoretical one-factor model, according to the performed factor analysis. Results of this study can potentially promote the initial mental health status assessment, usage of psychological therapy, and thus life quality enhancement.

Consistent with prior studies, our findings suggest that Vietnamese people were more likely to register their depression under the two-factor model instead of the one-factor of the original PHQ-9 (21, 40, 41). The first factor was “Somatic” with five items (Anhedonia, Depressed mood, Sleep disturbance, Fatigue, and Appetite changes) and the other was “Cognitive/Affective” with four items (Low Self-esteem, Concertation difficulties, Psychomotor disturbances, and Suicidal ideation). The unidimensional structure of the PHQ-9 has been corroborated in the majority of studies that look into its factor structure (26, 42–44). Other authors, on the other hand, suggest that the PHQ-9’s two-factor structure may be more interpretable in light of depression conceptual frameworks (20, 45, 46). The disparity between these research findings might be attributed to the participation of distinctive subgroups. Studies that indicated the superiority of the two-factor model, which is characterized by somatic and non-somatic (affective) symptoms, have been carried out on a number of clinical populations, namely those with concomitant physical conditions such as spinal cord injury (20) or cancer (45, 46). As a result, the somatic factor loading is probably induced by the confounding effects of physical and mental illness (43). However, Lamela et al. suggested that a two-factor model yielded fit in a general population sample (44), which is consistent with other empirical studies (36). Furthermore, our investigation found that for most people with moderate depression in a suburban environment, the two-factor model had greater fit indices compared to the theoretical one-factor from previous findings (36, 45). CFA results supported EFA findings when our two-factor model produced satisfactory fit indicators.

These two factors were given names based on the variables that made up the factors, as well as prior literature and the DSM-IV-TR (19). In comparison to the theoretical one-factor model used in earlier research, our two-factor model with factor 1 “Somatic” and factor 2 “Cognitive/Affective” had superior fit indices (40, 47) when two variables Q1 Anhedonia “Little interest or pleasure in doing things” and Q2 Depressed mood “Feeling gloomy, dejected, or hopeless” were added to factor 1 (40, 47). The cognitive-affective dimension of symptoms includes negative mood or negative affect, while the somatic dimension includes symptoms such as fatigue or loss of energy (48). Although several studies have shown that both two variables Q1 and Q2 suitable in factor 2 “Cognitive/Affective”, our findings showed that anhedonia or depressed mood could be characteristics to recognize somatic symptom disorder. Patients may experience a near-complete lack of happiness, desire, and interest in working because of these symptoms. However, more research is still needed to determine the items in each factor and the best PHQ-9 model for participants of different subgroups.

In addition, a significant link between the number of health problems and the risk of depression was found; this is consistent with the findings of previous studies (49, 50). People with health issues are concerned not only about their health but also about their family and socioeconomic status. In the context of accelerated urbanization, suburban people are often under financial pressure and in fear of getting ill since the expenditure on health care services can have detrimental effects on their financial health (51, 52).

Although only a small number of individuals reported smoking or alcohol consumption, those behaviors were linked to the somatic symptoms of depression. A possible rationale is that people tend to smoke or drink alcohol as a coping mechanism to deal with stress and depression (53). This consequently gives a potentially false impression of depressive symptoms relief (54, 55). The influence of stimulant usage on psychological discomfort, whether increased or lessened, is, nevertheless, still debatable. Moreover, in line with previous research, female gender is strongly associated with somatization (56–58); compared to males, females are more likely to report (59) somatic symptoms (e.g., eating disorder, headache, low body image confidence, insomnia, and exhaustion) in early adolescence (58, 60, 61).

In consonance with previous studies, moderate regular exercise can improve the cognitive and affective aspects of depression (62–64) and can aid in the prevention and cure of health problems such as high blood pressure, diabetes, and arthritis, and enhancing mood, eliminating anxiety, boosting confidence, and promoting social engagement (65, 66). In addition, people are likely to have reduced depressive symptoms if they obtain health information via radio/television, newspapers, health care workers or social network as these formal sources provide useful and reliable information about how to prevent such health problems and to raise awareness of health protection for themselves and their families (67–69).

A number of implications can be drawn from our study. Firstly, the PHQ-9 can be applied in clinical practice in Vietnam as a screening instrument. Potential patients in rural areas can fill the PHQ-9 before seeing a psychiatrist. Then the psychiatrist can use the scores to determine if the patient’s score is higher than the cut-off for depressive disorder, and it is determined that the symptoms are predominately somatic or cognitive/affective. If predominately somatic, the psychiatrists can consider providing psychotherapy to address somatic symptoms and provides symptomatic relief. In contrast, if the symptoms are predominately cognitive/affective and moderate to severe, the psychiatrists should consider antidepressants and cognitive training. This will enhance the efficiency of psychiatric services in Vietnam, which is characterized by a high volume of patient load. Severe depression can also be identified for more intense treatment. Furthermore, health and risk behavior education programs should be implemented to raise people’s health awareness of depression and how to treat it. Regulating health monitoring, encouraging people to exercise, and reducing substance use have a significant impact on the mental health of Vietnamese in suburban areas.

However, the limitation of this study should not be overlooked. The sampling may affect the generalizability of our findings. In order to demonstrate the consistency of the empirical findings, future studies should be performed on a larger and more diversified population. The inclusion of structured questionnaires like the one used in this study would allow for replication and could serve as a reference point for future research. In addition, our findings might be susceptible to recollection bias due to participants’ self-reporting process. Missing responses might also affect the result interpretation and reliability. Besides, the study did not use other reliability measures such as nomological validity and test-retest reliability. In addition, the need for a gold standard validation study, as well as a study exploring psychometric approaches such as item response theory, should be explored in future research in Vietnam. Lastly, prior research finds that the summed PHQ-9 has better diagnostic properties than the algorithm (70). In some studies, the application of the diagnostic algorithm score of the PHQ-9 as a method of validation includes the involvement of psychiatrists, and it is challenging for Vietnam as it is a developing country and does not have enough psychiatrists. In Vietnam, there are only 0.91 psychiatrists were available per 100,000 people. As a result, we do not have enough of a clinical workforce to use the PHQ-9 diagnostic algorithm.

## Conclusion

This study provided evidence that the PHQ-9 scale is a valid and internally reliable instrument to assess depression in the Vietnam population. The PHQ-9 has a stable two-factor structure. Both factors were found to be significantly associated with gender, health behavior, and health status in the suburban community in Vietnam. This scale can be a useful screening tool for depression, and further validation studies in other populations are recommended.

## Data Availability Statement

The original contributions presented in this study are included in the article/supplementary material, further inquiries can be directed to the corresponding author.

## Ethics Statement

This research project was reviewed and managed by the Vietnam Ministry of Health under Decision 850/QD-BYT. Ethical approval was granted by the IRB committee at Hanoi Medical University and the Ministry of Health. The patients/participants provided their written informed consent to participate in this study.

## Author Contributions

TV, TL, CL, and CH: conceptualization. ADa, LL, SN, ADo, and ND: data curation. LV, ADa, SN, and TV: formal analysis. SN, TV, TL, RH, and CH: investigation. ADa, LL, TT, ADo, and ND: methodology. TT, RH, and CH: supervision. LV, TT, ADo, TL, and CL: writing – original draft. LV, LL, ND, TL, CL, and RH: writing – review and editing. All authors contributed to the article and approved the submitted version.

## Conflict of Interest

The authors declare that the research was conducted in the absence of any commercial or financial relationships that could be construed as a potential conflict of interest.

## Publisher’s Note

All claims expressed in this article are solely those of the authors and do not necessarily represent those of their affiliated organizations, or those of the publisher, the editors and the reviewers. Any product that may be evaluated in this article, or claim that may be made by its manufacturer, is not guaranteed or endorsed by the publisher.

## References

[1] World Health Organization [WHO]. *Depression and Other Common Mental Disorders: Global Health Estimates.* Geneva: World Health Organization (2017).

[2] Belmaker RH Agam G. Major depressive disorder. *N Engl J Med.* (2008) 358:55–68. 10.1056/NEJMra073096 18172175

[3] Otte C Gold SM Penninx BW Pariante CM Etkin A Fava M Major depressive disorder. *Nat Rev Dis Primers.* (2016) 2:16065. 10.1038/nrdp.2016.65 27629598

[4] Isometsa ET Henriksson MM Aro HM Heikkinen ME Kuoppasalmi KI Lonnqvist JK. Suicide in major depression. *Am J Psychiatry.* (1994) 151:530–6. 8147450 10.1176/ajp.151.4.530

[5] Hesdorffer DC Hauser WA Olafsson E Ludvigsson P Kjartansson O. Depression and suicide attempt as risk factors for incident unprovoked seizures. *Ann Neurol.* (2006) 59:35–41. 10.1002/ana.20685 16217743

[6] Harpham T. Urban health in developing countries: What do we know and where do we go? *Health Place.* (2009) 15:107–16. 10.1016/j.healthplace.2008.03.004 18455952

[7] Elgar FJ Arlett C Groves R. Stress, coping, and behavioural problems among rural and urban adolescents. *J Adolesc.* (2003) 26:577–88. 10.1016/s0140-1971(03)00057-5 12972270

[8] Chen J Chen S Landry PF. Urbanization and mental health in China: linking the 2010 population census with a cross-sectional survey. *Int J Environ Res Public Health.* (2015) 12:9012–24. 10.3390/ijerph120809012 26264013 PMC4555260

[9] Kawachi I Kennedy BP Glass R. Social capital and self-rated health: a contextual analysis. *Am J Public Health.* (1999) 89:1187–93. 10.2105/AJPH.89.8.1187 10432904 PMC1508687

[10] Ellwardt L Aartsen M Deeg D Steverink N. Does loneliness mediate the relation between social support and cognitive functioning in later life? *Soc Sci Med.* (2013) 98:116–24. 10.1016/j.socscimed.2013.09.002 24331889

[11] Boneham MA Sixsmith JA. The voices of older women in a disadvantaged community: issues of health and social capital. *Soc Sci Med.* (2006) 62:269–79. 10.1016/j.socscimed.2005.06.003 16039027

[12] Harpham T. Urbanization and mental health in developing countries: a research role for social scientists, public health professionals and social psychiatrists. *Soc Sci Med.* (1994) 39:233–45. 10.1016/0277-9536(94)90332-8 8066502

[13] Zhang L Wang SX Yu L. Is social capital eroded by the state-led urbanization in China? A case study on indigenous villagers in the urban fringe of Beijing. *China Econ Rev.* (2015) 35:232–46. 10.1016/j.chieco.2014.04.005

[14] Spitzer RL Kroenke K Williams JB. Validation and utility of a self-report version of PRIME-MD: the PHQ primary care study. Primary care evaluation of mental disorders. Patient health questionnaire. *JAMA.* (1999) 282:1737–44. 10.1001/jama.282.18.1737 10568646

[15] Wittkampf KA Naeije L Schene AH Huyser J van Weert HC. Diagnostic accuracy of the mood module of the patient health questionnaire: a systematic review. *Gen Hosp Psychiatry.* (2007) 29:388–95. 10.1016/j.genhosppsych.2007.06.004 17888804

[16] Manea L Gilbody S McMillan D. Optimal cut-off score for diagnosing depression with the Patient Health Questionnaire (PHQ-9): a meta-analysis. *CMAJ.* (2012) 184:E191–6. 10.1503/cmaj.110829 22184363 PMC3281183

[17] Kroenke K Spitzer RL Williams JB Lowe B. The patient health questionnaire somatic, anxiety, and depressive symptom scales: a systematic review. *Gen Hosp Psychiatry.* (2010) 32:345–59. 10.1016/j.genhosppsych.2010.03.006 20633738

[18] Kroenke K Spitzer RL Williams JB. The PHQ-9: validity of a brief depression severity measure. *J Gen Intern Med.* (2001) 16:606–13. 10.1046/j.1525-1497.2001.016009606.x 11556941 PMC1495268

[19] American Psychiatric Association [APA]. *Diagnostic and Statistical Manual of Mental Disorders.* (Vol. 3). Washington, DC: American Psychiatric Association (1980).

[20] Beard C Hsu KJ Rifkin LS Busch AB Bjorgvinsson T. Validation of the PHQ-9 in a psychiatric sample. *J Affect Disord.* (2016) 193:267–73. 10.1016/j.jad.2015.12.075 26774513

[21] Petersen JJ Paulitsch MA Hartig J Mergenthal K Gerlach FM Gensichen J. Factor structure and measurement invariance of the Patient Health Questionnaire-9 for female and male primary care patients with major depression in Germany. *J Affect Disord.* (2015) 170:138–42. 10.1016/j.jad.2014.08.053 25240840

[22] de Jonge P Mangano D Whooley MA. Differential association of cognitive and somatic depressive symptoms with heart rate variability in patients with stable coronary heart disease: findings from the Heart and Soul Study. *Psychosom Med.* (2007) 69:735–9. 10.1097/PSY.0b013e31815743ca 17942844 PMC2776660

[23] Krause JS Reed KS McArdle JJ. Factor structure and predictive validity of somatic and nonsomatic symptoms from the patient health questionnaire-9: a longitudinal study after spinal cord injury. *Arch Phys Med Rehabil.* (2010) 91:1218–24. 10.1016/j.apmr.2010.04.015 20684902

[24] Liu SI Yeh ZT Huang HC Sun FJ Tjung JJ Hwang LC Validation of patient health questionnaire for depression screening among primary care patients in Taiwan. *Compr Psychiatry.* (2011) 52:96–101. 10.1016/j.comppsych.2010.04.013 21111406

[25] Yu X Tam WW Wong PT Lam TH Stewart SM. The Patient Health Questionnaire-9 for measuring depressive symptoms among the general population in Hong Kong. *Compr Psychiatry.* (2012) 53:95–102. 10.1016/j.comppsych.2010.11.002 21193179

[26] Kocalevent RD Hinz A Brahler E. Standardization of the depression screener patient health questionnaire (PHQ-9) in the general population. *Gen Hosp Psychiatry.* (2013) 35:551–5. 10.1016/j.genhosppsych.2013.04.006 23664569

[27] Adewuya AO Ola BA Afolabi OO. Validity of the patient health questionnaire (PHQ-9) as a screening tool for depression amongst Nigerian university students. *J Affect Disord.* (2006) 96:89–93. 10.1016/j.jad.2006.05.021 16857265

[28] Anum A Adjorlolo S Kugbey N. Depressive symptomatology in adolescents in Ghana: examination of psychometric properties of the patient health questionnaire-9. *J Affect Disord.* (2019) 256:213–8. 10.1016/j.jad.2019.06.007 31181377

[29] Granillo MT. Structure and function of the patient health questionnaire-9 among Latina and non-Latina white female college students. *J Soc Soc Work Res.* (2012) 3:80–93. 10.5243/jsswr.2012.6

[30] Do HN Nguyen AT Nguyen HQT Bui TP Nguyen QV Tran NTT Depressive symptoms, suicidal ideation, and mental health service use of industrial workers: evidence from Vietnam. *Int J Environ Res Public Health.* (2020) 17:2929. 10.3390/ijerph17082929 32340335 PMC7216084

[31] Nguyen Hang Nguyet V Nguyen Thi Khanh H Nguyen Thanh L Duong Minh D Pham Quoc T. Factors associated with depression among the elderly living in rural Vietnam 2019: recommendations to remove barriers of psychological service accessibility. *Int J Ment Health.* (2021) 50:136–50. 10.1080/00207411.2020.1855050

[32] Luong-Thanh BY Nguyen LH Murray L Eisner M Valdebenito S Hoang TD Depression and its associated factors among pregnant women in central Vietnam. *Health Psychol Open.* (2021) 8:2055102920988445. 10.1177/2055102920988445 33598304 PMC7841685

[33] Carey M Boyes A Noble N Waller A Inder K. Validation of the PHQ-2 against the PHQ-9 for detecting depression in a large sample of Australian general practice patients. *Aust J Prim Health.* (2016) 22:262–6. 10.1071/PY14149 26306421

[34] Cheung K Oemar M Oppe M Rabin R. *EQ-5D User Guide: Basic Information on How to Use EQ-5D.* Rotterdam: EuroQol Group (2009).

[35] Tran BX Ohinmaa A Nguyen LT Nguyen TA Nguyen TH. Determinants of health-related quality of life in adults living with HIV in Vietnam. *AIDS Care.* (2011) 23:1236–45. 10.1080/09540121.2011.555749 21711211

[36] Godin G Shephard RJ. Godin leisure-time exercise questionnaire. *Med Sci Sports.* (1997) 29:S36–8. 10.1097/00005768-199706001-00009

[37] Tavakol M Dennick R. Making sense of Cronbach’s alpha. *Int J Med Educ.* (2011) 2:53–5. 10.5116/ijme.4dfb.8dfd 28029643 PMC4205511

[38] Ledesma RD Valero-Mora P. Determining the number of factors to retain in EFA: an easy-to-use computer program for carrying out parallel analysis. *Pract Assess Res Eval.* (2007) 12:2.

[39] Hooper D Coughlan J Mullen M. Structural equation modeling: guidelines for determining model fit. *Electr J Bus Res Methods.* (2007) 6:53–60.

[40] Patel JS Oh Y Rand KL Wu W Cyders MA Kroenke K Measurement invariance of the patient health questionnaire-9 (PHQ-9) depression screener in U.S. adults across sex, race/ethnicity, and education level: NHANES 2005-2016. *Depress Anxiety.* (2019) 36:813–23. 10.1002/da.22940 31356710 PMC6736700

[41] Vrany EA Berntson JM Khambaty T Stewart JC. Depressive symptoms clusters and insulin resistance: race/ethnicity as a moderator in 2005-2010 NHANES data. *Ann Behav Med.* (2016) 50:1–11. 10.1007/s12160-015-9725-0 26318593

[42] Titov N Dear BF McMillan D Anderson T Zou J Sunderland M. Psychometric comparison of the PHQ-9 and BDI-II for measuring response during treatment of depression. *Cogn Behav Ther.* (2011) 40:126–36. 10.1080/16506073.2010.550059 25155813

[43] Erbe D Eichert HC Rietz C Ebert D. Interformat reliability of the patient health questionnaire: validation of the computerized version of the PHQ-9. *Internet Interv.* (2016) 5:1–4. 10.1016/j.invent.2016.06.006 30135800 PMC6096192

[44] Ryan TA Bailey A Fearon P King J. Factorial invariance of the patient health questionnaire and generalized anxiety disorder questionnaire. *Br J Clin Psychol.* (2013) 52:438–49. 10.1111/bjc.12028 24117915 PMC4296344

[45] Hinz A Mehnert A Kocalevent RD Brahler E Forkmann T Singer S Assessment of depression severity with the PHQ-9 in cancer patients and in the general population. *BMC Psychiatry.* (2016) 16:22. 10.1186/s12888-016-0728-6 26831145 PMC4736493

[46] Chilcot J Rayner L Lee W Price A Goodwin L Monroe B The factor structure of the PHQ-9 in palliative care. *J Psychosom Res.* (2013) 75:60–4. 10.1016/j.jpsychores.2012.12.012 23751240

[47] Doi S Ito M Takebayashi Y Muramatsu K Horikoshi M. Factorial validity and invariance of the Patient Health Questionnaire (PHQ)-9 among clinical and non-clinical populations. *PLoS One.* (2018) 13:e0199235. 10.1371/journal.pone.0199235 30024876 PMC6053131

[48] Kapfhammer HP. Somatic symptoms in depression. *Dialogues Clin Neurosci.* (2006) 8:227–39. 10.31887/DCNS.2006.8.2/hpkapfhammer 16889108 PMC3181769

[49] Zhang Y Chen Y Ma L. Depression and cardiovascular disease in elderly: current understanding. *J Clin Neurosci.* (2018) 47:1–5. 10.1016/j.jocn.2017.09.022 29066229

[50] Agborsangaya CB Lau D Lahtinen M Cooke T Johnson JA. Health-related quality of life and healthcare utilization in multimorbidity: results of a cross-sectional survey. *Qual Life Res.* (2013) 22:791–9. 10.1007/s11136-012-0214-7 22684529

[51] Dutt AK Noble AG Venugopal G Subbiah S. *Challenges to Asian Urbanization in the 21st Century.* Berlin: Springer Science & Business Media (2003).

[52] Wagner LN. *Urbanization: 21st Century Issues and Challenges.* Hauppauge, NY: Nova Publishers (2008).

[53] Rosario M Schrimshaw EW Hunter J. Cigarette smoking as a coping strategy: negative implications for subsequent psychological distress among lesbian, gay, and bisexual youths. *J Pediatr Psychol.* (2011) 36:731–42. 10.1093/jpepsy/jsp141 20123704 PMC3146751

[54] Kassel JD Evatt DP Greenstein JE Wardle MC Yates MC Veilleux JC. The acute effects of nicotine on positive and negative affect in adolescent smokers. *J Abnorm Psychol.* (2007) 116:543–53. 10.1037/0021-843X.116.3.543 17696710

[55] Quello SB Brady KT Sonne SC. Mood disorders and substance use disorder: a complex comorbidity. *Sci Pract Perspect.* (2005) 3:13–21. 10.1151/spp053113 18552741 PMC2851027

[56] Silverstein B. Gender difference in the prevalence of clinical depression: the role played by depression associated with somatic symptoms. *Am J Psychiatry.* (1999) 156:480–2. 10.1176/ajp.156.3.480 10080570

[57] Silverstein B. Gender differences in the prevalence of somatic versus pure depression: a replication. *Am J Psychiatry.* (2002) 159:1051–2. 10.1176/appi.ajp.159.6.1051 12042198

[58] Wenzel A Steer RA Beck AT. Are there any gender differences in frequency of self-reported somatic symptoms of depression? *J Affect Disord.* (2005) 89:177–81. 16202455 10.1016/j.jad.2005.06.009

[59] Sandanger I Nygard JF Sorensen T Moum T. Is women’s mental health more susceptible than men’s to the influence of surrounding stress? *Soc Psychiatry Psychiatr Epidemiol.* (2004) 39:177–84. 10.1007/s00127-004-0728-6 14999449

[60] Bjornelv S Nordahl HM Holmen TL. Psychological factors and weight problems in adolescents. The role of eating problems, emotional problems, and personality traits: the Young-HUNT study. *Soc Psychiatry Psychiatr Epidemiol.* (2011) 46:353–62. 10.1007/s00127-010-0197-z 20238097

[61] Bennett DS Ambrosini PJ Kudes D Metz C Rabinovich H. Gender differences in adolescent depression: do symptoms differ for boys and girls? *J Affect Disord.* (2005) 89:35–44. 16219362 10.1016/j.jad.2005.05.020

[62] Paolucci EM Loukov D Bowdish DME Heisz JJ. Exercise reduces depression and inflammation but intensity matters. *Biol Psychol.* (2018) 133:79–84. 29408464 10.1016/j.biopsycho.2018.01.015

[63] Schuch FB Vancampfort D Sui X Rosenbaum S Firth J Richards J Are lower levels of cardiorespiratory fitness associated with incident depression? A systematic review of prospective cohort studies. *Prev Med.* (2016) 93:159–65. 27765659 10.1016/j.ypmed.2016.10.011

[64] Harvey SB Hotopf M Overland S Mykletun A. Physical activity and common mental disorders. *Br J Psychiatry.* (2010) 197:357–64. 21037212 10.1192/bjp.bp.109.075176

[65] Cooney GM Dwan K Greig CA Lawlor DA Rimer J Waugh FR Exercise for depression. *Cochrane Database Syst Rev.* (2013) 9:CD004366. 10.1002/14651858.CD004366.pub6 PMC9721454 24026850

[66] Rosenbaum S Tiedemann A Sherrington C Curtis J Ward PB. Physical activity interventions for people with mental illness: a systematic review and meta-analysis. *J Clin Psychiatry.* (2014) 75:964–74. 24813261 10.4088/JCP.13r08765

[67] Al-Garadi MA Khan MS Varathan KD Mujtaba G Al-Kabsi AM. Using online social networks to track a pandemic: a systematic review. *J Biomed Inform.* (2016) 62:1–11. 10.1016/j.jbi.2016.05.005 27224846

[68] Fung IC Tse ZT Fu KW. The use of social media in public health surveillance. *Western Pac Surveill Response J.* (2015) 6:3–6. 10.5365/wpsar.2015.6.1.019 26306208 PMC4542478

[69] Yan Q Tang S Gabriele S Wu J. Media coverage and hospital notifications: correlation analysis and optimal media impact duration to manage a pandemic. *J Theor Biol.* (2016) 390:1–13. 10.1016/j.jtbi.2015.11.002 26582723

[70] Manea L Gilbody S McMillan D. A diagnostic meta-analysis of the Patient Health Questionnaire-9 (PHQ-9) algorithm scoring method as a screen for depression. *Gen Hosp Psychiatry.* (2015) 37:67–75. 10.1016/j.genhosppsych.2014.09.009 25439733

